# A Parametric Approach to Shape Field-Relevant Blast Wave Profiles in Compressed-Gas-Driven Shock Tube

**DOI:** 10.3389/fneur.2014.00253

**Published:** 2014-12-02

**Authors:** Aravind Sundaramurthy, Namas Chandra

**Affiliations:** ^1^Center for Injury Bio-Mechanics, Materials and Medicine, New Jersey Institute of Technology, Newark, NJ, USA

**Keywords:** primary blast injury, explosion modeling, shock tube, blast induced neurotrauma, shock tube adjustable parameters, shock wave parameters

## Abstract

Detonation of a high-explosive produces shock-blast wave, shrapnel, and gaseous products. While direct exposure to blast is a concern near the epicenter, shock-blast can affect subjects, even at farther distances. When a pure shock-blast wave encounters the subject, in the absence of shrapnels, fall, or gaseous products the loading is termed as *primary blast loading* and is the subject of this paper. The wave profile is characterized by blast overpressure, positive time duration, and impulse and called herein as shock-blast wave parameters (SWPs). These parameters in turn are uniquely determined by the strength of high explosive and the distance of the human subjects from the epicenter. The shape and magnitude of the profile determine the severity of injury to the subjects. As shown in some of our recent works (1–3), the profile not only determines the survival of the subjects (e.g., animals) but also the acute and chronic biomechanical injuries along with the following bio-chemical sequelae. It is extremely important to carefully design and operate the shock tube to produce field-relevant SWPs. Furthermore, it is vital to identify and eliminate the artifacts that are inadvertently introduced in the shock-blast profile that may affect the results. In this work, we examine the relationship between shock tube adjustable parameters (SAPs) and SWPs that can be used to control the blast profile; the results can be easily applied to many of the laboratory shock tubes. Further, replication of shock profile (magnitude and shape) can be related to field explosions and can be a standard in comparing results across different laboratories. Forty experiments are carried out by judiciously varying SAPs such as membrane thickness, breech length (66.68–1209.68 mm), measurement location, and type of driver gas (nitrogen, helium). The effects SAPs have on the resulting shock-blast profiles are shown. Also, the shock-blast profiles of a TNT explosion from ConWep software is compared with the profiles obtained from the shock tube. To conclude, our experimental results demonstrate that a compressed-gas shock tube when designed and operated carefully can replicate the blast time profiles of field explosions accurately. Such a faithful replication is an essential first step when studying the effects of blast induced neurotrauma using animal models.

## Introduction

In the study of blast induced neurotrauma (BINT) using animal models, many research groups use compressed-gas-driven shock tubes to simulate primary blast injury conditions (4–9). Since the injury to animals critically depends on the nature of the shock-blast wave (from here on simply known as blast waves), it is important to standardize the blast wave across the various shock tubes. Though the generation of shock wave by itself is straightforward, controlling the shape and magnitude of the pressure-time pulse is not trivial, and is subject of the present paper.

In general, detonation of a high explosive on or near surface produces blast wave, noise, shrapnel, and toxic gaseous products. Blast (the air shock wave due to explosion) is a major concern due to its ability to cause damage at relatively long distances from the point of explosion (10, 11). Numerous cases of soldier injuries in operation Iraqi freedom (OIF) and operation enduring freedom (OEF) were directly attributed to the blasts, resulting from improvised explosive devices (IEDs) used by the insurgents. Among these injuries, the most common was the traumatic brain injury, which is the signature injury of these conflicts (12–15). In a study conducted by RAND Corporation, it was estimated that 320,000 service members or 20% of the deployed force (total deployed 1.6 million) potentially suffer from TBI (includes primary, secondary, tertiary, and quaternary injuries); however, out of this population, approximately 60% have never been assessed by a healthcare provider specifically for TBI and suffer from mild TBI (16). It should be noted that the blunt impacts (included in tertiary injury) can be ascribed to the blast events (e.g., mounted soldiers inside the vehicle subjected to IEDs); however, all the blunt impacts may or may not be related to blast events. The mild TBI is classified as loss of consciousness for <30 min or amnesia lasting <24 h, and is not detectable during early stages post-injury using any of the current neuroimaging techniques.

Recently, an extensive research effort has been initiated toward the understanding of the mechanism of primary blast injury. Initial findings from the experimental models and the computer simulations suggest that most of these injuries may have been due to the direct impingement of the blast wave on the skull (3, 6, 8, 17–21). Other mechanisms including thoracic pressure surge, skull deflection, and cavitation are also being explored as the loading mechanisms. Although, the free-field blast testing closely replicates real-world blast conditions, there are some significant drawbacks: (i) free-field experiments are expensive and unsafe; (ii) time consuming; (iii) repeatability is difficult to achieve, as it is difficult to control the environment of the field blast with byproducts that include fireball interactions and penetration from high-rate shrapnel (15). This can also introduce unnecessary confounds to the experiments where the objective is to understand the mechanisms of primary blast injury and its subsequent biomechanical and bio-chemical sequelae. A recent review article by Kobeissy and his colleagues shows that out of 49 studies, only 8 used field testing and 92% of shock tubes (33 out of 36) used were compressed-gas-driven shock tubes (Table 1) (22).

**Table 1:** **Explosive capacity of the currently used IEDs and mines in the field**.

Threat	Explosive capacity (TNT equivalent in kg)	Reference
Pipe bomb	2.28	(23)
Suicide bomber	9	
Briefcase bomb	22.70	
AP fragmentation device	0.55	(24)
AV blast landmine	6–10	

*Explosive capacities of these IEDs are given in terms of TNT equivalents in kilograms*.

**Figure 1:**
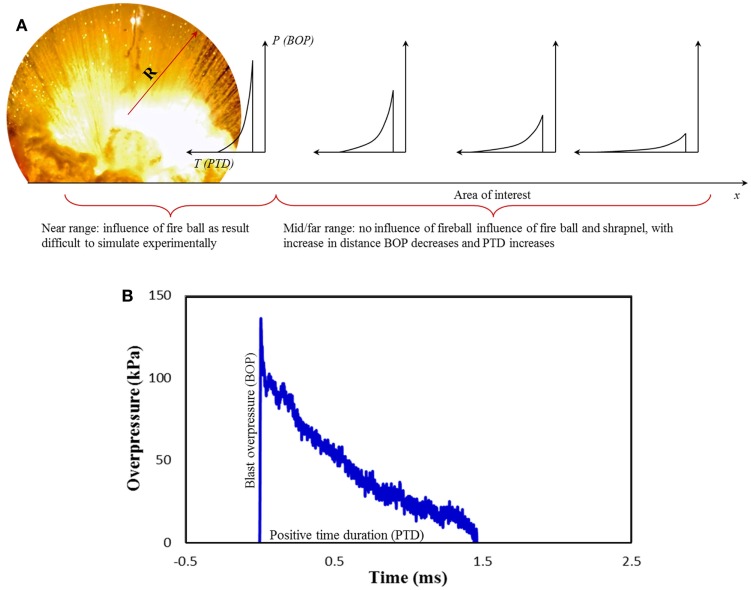
**(A)** shows evolution of shock-blast profile as the distance from the epicenter increases. Radius from epicenter with BOP higher than 1000 kPa is considered far range, which is very close to the fireball (R) and outside this radius it is mid and far field range, **(B)** Shock-blast wave profile generated from the explosion of 1.814 kg of C_4_ at a distance of 2.8 m.

Accurately, simulating blast wave in laboratory condition using a compressed-gas shock tube requires the ability to control shock-blast wave parameters (SWPs) independently to mimic a field explosion. For a simplified hemispherical explosion as shown in Figure 1A, depending on the length of the fireball radius, the blast is divided into three regimes, near field, mid field, and far field (25). Objects that are exposed to incident pressure of 1000 kPa or higher are typically within/near the fireball and considered as a near field condition. It is said within the near field condition the following is expected: (a) interaction with detonation products/shrapnel; (b) complex evolution of the waveforms; and (c) high gradients in the flow of temperature and density. In reality, flow conditions in the near field for IEDs are lot more complex due to the irregular charge shape, casing/shrapnel (where some of the kinetic energy of the blast is used for accelerating the shrapnel from the casing), and buried/grounded. It is extremely difficult to device a methodology to simulate the near field condition due to its sheer unstable nature. These extreme conditions cause very severe injuries and immediate death. Following the near field is the mid field and far field, which is referred as the zone beyond fireball. Pressure profiles in the mid and far field have a similar shape with amplitude decaying monotonically and duration increasing with increasing distance (Figure 1A). Eventually, the wave loses its strength (i.e., Mach number <1) and decays into a sound wave (26). Figure 1B shows typical pressure-time profile recorded from a field explosion of 1.814 kg (4 lb) of C_4_ at a distance of 2.8 m from the epicenter of the blast measured using a PCB press type pencil gage (model: 137A22). From the figure, it can be seen that there is a sudden rise in the pressure, which represents the shock front (i.e., blast overpressure) followed by an exponential decay. This sudden rise followed by an exponential decay comprises the positive phase of the blast wave, which can be described using a modified Friedlander equation.

$$p\left(t\right)=p_{o}\left(1-\frac{t}{t_{d}}\right)e^{\left(\frac{-t}{\alpha }\right)}$$

where *p_o_* represents the blast overpressure, *t_d_* represents the positive time duration (PTD), and α represents the time decay constant (26). Although, this exponential decay reaches sub-atmospheric pressures generating a negative phase, Friedlander model does not take that into account (27). Obtaining a Friedlander type profile (sharp rise-exponential decay) does not guarantee realistic shock profile as the parameters (e.g., duration and impulse) may not match any combination of explosive strength and stand-off distance. In addition, in actual field explosions the shape of the curve may be complex, determined by the shape, size, and type of explosives; sub-surface burial depth; temperature, altitude, wind, and humidity of the environment; and presence/absence of enclosures and obstructions. However, a blast wave of Friedlander type can still be used as the basic building block to model the complex input condition, as followed by most researchers.

In this work, we are examining the role of different geometric, constructional, and operational features (such as breech length, type of gas, membrane thickness, and measurement location) of compressed-gas-driven shock tubes has on the SWPs (such as blast overpressure and PTD). We also characterize the flat top or plateau wave and determine the influence driver gas and breech length have on the blast profiles. Finally, we compare the blast wave profile from the shock tube with the field explosion profiles generated in ConWep (Conventional Weapons effects). With this, we hope to standardize the method for generating blast pulses so that the bio-chemical, biomechanical, and medical results obtained across various groups can be compared and correlated among themselves and with the field data.

## Materials and Methods

**Figure 2:**
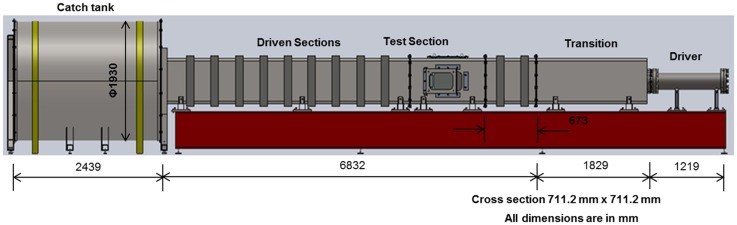
**711 mm × 711 mm Shock tube system**. (Note: compressed gas is pumped into driver section in the right and the shock propagates from right to left in the figure.)

Experiments were carried out in the shock tube designed by our group and tested at the University of Nebraska-Lincoln’s blast wave generation facility (Figure 2) (1) (a similar system has been developed in Center for injury biomechanics, materials, and medicine at NJIT). The four main components of any compressed-gas-driven shock tube are driver, transition, driven sections, and catch tank. The driver section (breech) contains pressurized gas (e.g., nitrogen, helium), which is separated from the transition by several frangible Mylar^®^ membranes, while the driven section (including the expansion section) contains air at atmospheric pressure and room temperature. Transition section acts as an adapter, which is used to change the cross-section of the tube from a circular (breech) to a square (expansion section); the square section is a design element to observe events in the test section with high-speed video imaging. The driven section has a 711.2 mm × 711.2 mm (28″ × 28″), cross-section, and the length of 8661 mm. Finally, catch tank absorbs the kinetic energy of the exiting jet from the shock tube and does not play any role in modifying the profile. Upon membrane rupture, a blast wave is generated, which expands through the transition and develops into planar blast waveform in the driven section. Driven section also encompasses the test section. Finally, the blast wave exits the shock tube and enters the catch tank, which reduces the noise intensity. The cross-sectional dimension of this shock tube is designed such that subjects within the test section experiences a planar blast wave without significant sidewall reflections. The planarity of the blast wave is verified by pressure measurements across the test section of the shock tube (28).

**Figure 3:**
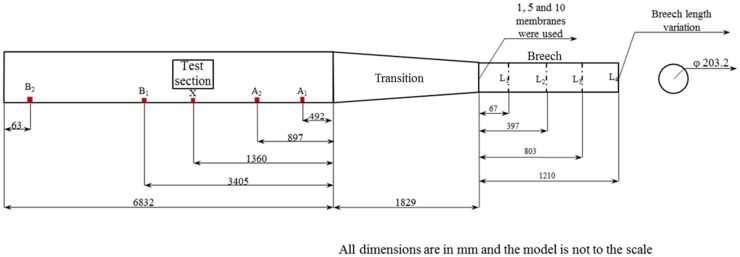
**Experimental variables and sensor location; here, A1, A2, X, B1, and B2 are the side-on pressure sensors**. Here, L_1_, L_2_, L_3_, and L_4_ are the breech lengths used in the experiment, A_1_, A_2_, X, B_1_, and B_2_ are the incident pressure sensor locations and 1, 5, and 10 are the number of membranes used.

Figure 3 shows the experimental variables and the sensor locations. The length of the breech is varied with discrete increments designated as L_1_ (66.68 mm), L_2_ (396.88 mm), L_3_ (803.28 mm), and L_4_ (1209.68 mm). The membrane thickness is varied by varying the number of membranes between 1, 5, and 10 (each membrane is 0.254 mm thick). In this work, both nitrogen and helium were used as the driver gas, and the driven gas was air at ambient laboratory conditions (temperature range of 23 ± 2° C). The evolution of the blast wave along the length of the shock tube was measured using PCB pressure gages (model 134A24) mounted on the wall of the shock tube at locations A1, A2, X, B1, and B2 (Figure 3). Burst pressure in driver just before the rupture of the membranes was also recorded.

## Results

### Burst pressure

**Figure 4:**
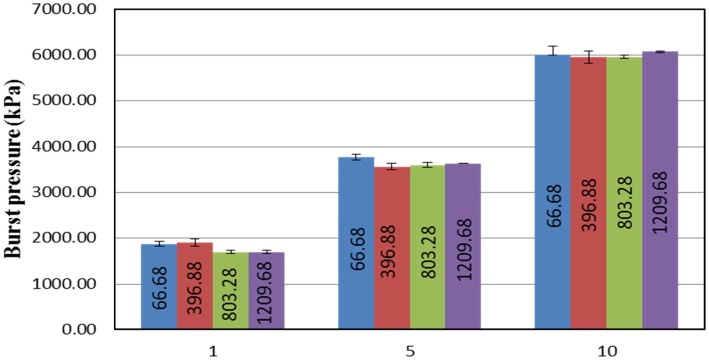
**Relationship between the number of membranes used and burst pressure produced with respect to different breech lengths**. From this figure, it can be seen that there is no significant difference in the burst pressure with respect to the variations in the breech length. However, the burst pressure tends to increase with an increase in the number of membranes used.

Burst pressure is the pressure in the driver section (breech) at the time of the membrane rupture. This highly compressed gas when allowed to expand rapidly compresses the atmospheric air in the transition and driven sections generating a shock front. Burst pressure for different membrane thicknesses and breech lengths are shown in Figure 4. The burst pressure increases with an increase in the membrane thickness. There is no discernible difference in the burst pressure with respect to increase in breech length for any of the three membrane thicknesses studied. It should be noted that any variation in the burst pressure for identical conditions (e.g., number of membranes, breech volume, and type of gas) will be due to the variations in filling rate of gas in the breech; since Mylar^®^ membrane is viscoelastic in nature the rate of deformation depends on the rate of pressurization of the driver section. In our experience, there is a pronounced rate dependency effect for smaller breech lengths (results not shown for the sake of brevity), compared to larger ones.

### Shock tube adjustable parameters and their influence on the blast wave parameters in test section

**Figure 5:**
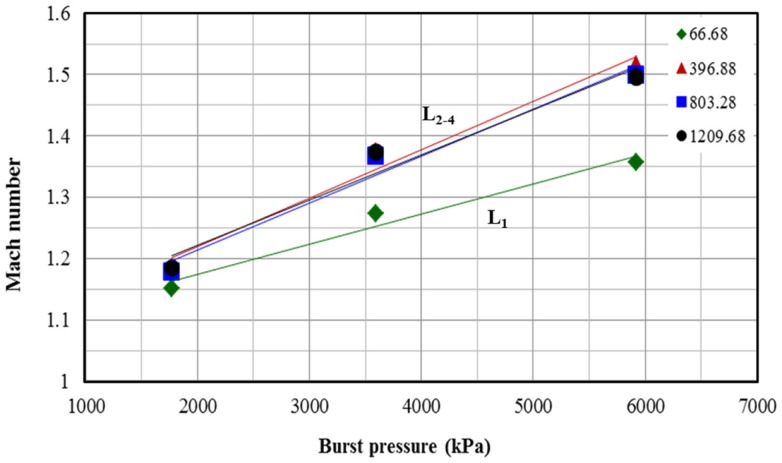
**Relationship between shock front Mach number and burst pressure for different breech lengths, there is linear relationship between Mach number and burst pressure (i.e., increase in membrane thickness)**.

By changing the shock tube adjustable parameters (SAPs) such as membrane thickness (burst pressure) and breech length, we can alter the SWPs such as Mach number, blast overpressure, and PTD in the test section. Mach number of the shock front refers to the ratio of the velocity of the shock in the given medium to the velocity of sound in the same medium. From Figure 5, it can be seen that the Mach number of the shock front depends on the burst pressure and has a positive linear relationship with burst pressure, i.e., shock front velocity increases with an increase in the burst pressure. When breech length is L_1_ (small) Mach number increases at a lower rate (slope of the line) compared to that of the other breech lengths (L_2_, L_3_, and L_4_). How and why the behavior for shorter breech length is different from that of the others will be discussed later.

**Figure 6:**
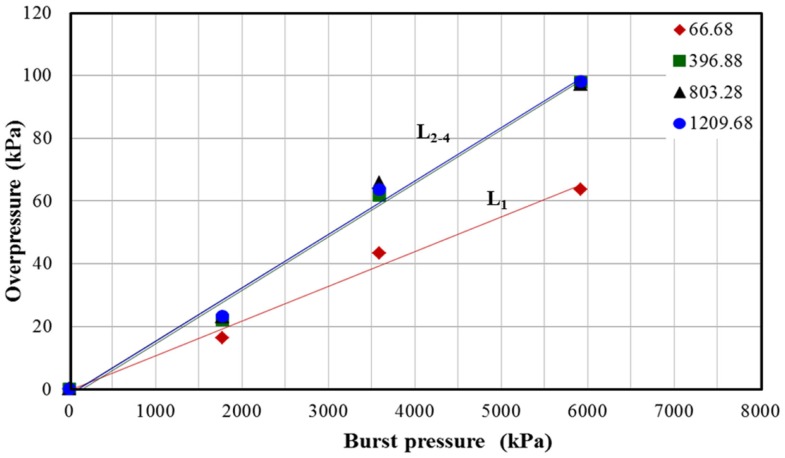
**Relationship between shock tube parameter burst pressure with overpressure measured in the test section for different breech lengths**.

Blast overpressure is the gage pressure measured in the air, which is the difference between absolute gas pressure and atmospheric pressure. Similar to Mach number, there is a linear relationship between blast overpressure and burst pressure (Figure 6). With increase in the burst pressure the blast overpressure for L_2_, L_3_, and L_4_ increases with a higher rate (higher slope) compared to that of L_1_. These results are intriguing and are explained in the discussion section.

**Figure 7:**
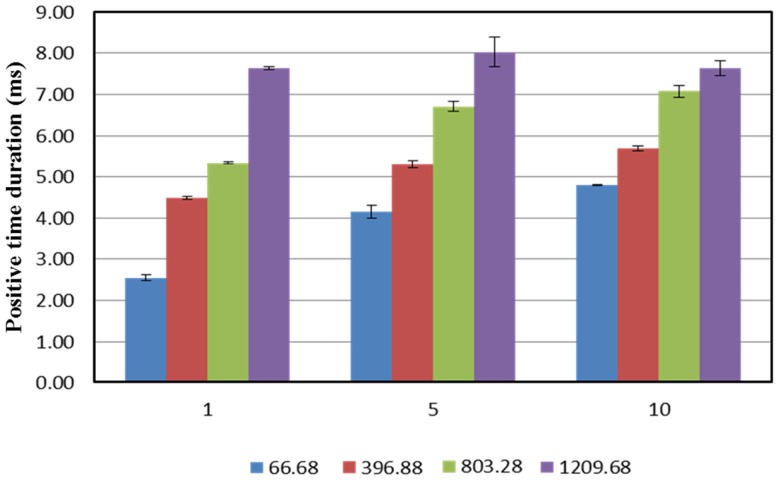
**Relationship between positive time duration (PTD) and membrane thickness used for different breech lengths**. It can be seen that there PTD increases with increase in the breech length for any given membrane configuration; furthermore, for a lower breech lengths, the PTD tends to increase with increase in the number of membranes used, however, this change reduces with increase in breech length. For the maximum breech length, there is no significant change in the PTD.

Positive time duration is the period when the blast overpressure reduces to 0, i.e., when it reaches the local atmospheric pressure. From Figure 7, it can be seen that for a given membrane thickness, PTD increases with an increase in the breech length. There is an increase in PTD between membrane thicknesses 1 and 5 for breech lengths L_1_, L_2_, and L_3_; however, such an apparent difference is not observed between membrane thicknesses 5 and 10. Finally, for breech length L_4_ there is no apparent difference in PTD for different membrane thicknesses.

### Evolution of the blast wave along the expansion section

**Figure 8:**
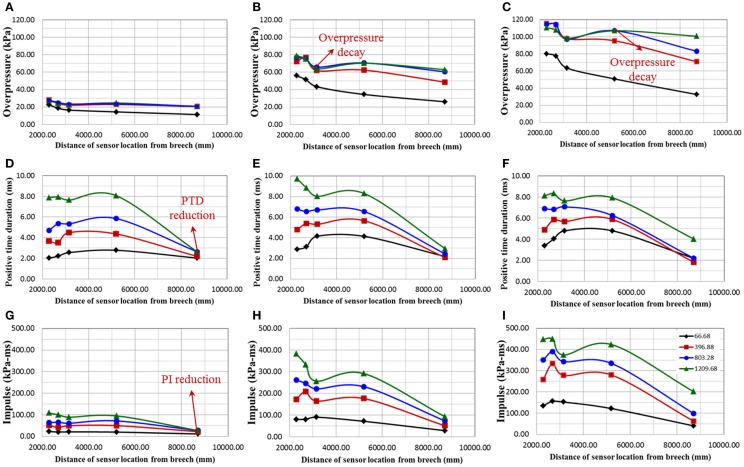
**Describes the variation of shock-blast profile parameters along the length of the shock tube expansion section; all these experiments were performed for breech lengths 66.68 (black), 396.88 (red), 803.28 (blue), and 1209.68 (green) mm**. **(A–C)** show the variation of overpressure along the length of the expansion section for burst pressures corresponding to 1, 5, and 10 membranes, respectively; **(D–F)** show the positive time duration along the expansion section for burst pressures corresponding to 1, 5, and 10 membranes, respectively; **(G–I)** show the positive impulse along the expansion section for burst pressures corresponding to 1, 5, and 10 membranes, respectively.

Figures 8A–C show the evolution of the blast overpressure along the length of the expansion section. From Figure 8A, it can be seen that, for one membrane there is no discernible change in blast overpressure for breech lengths L_2_, L_3_, and L_4_. For all cases with breech length L_1_, there is a continuous decay in the blast overpressure downstream of the shock tube. For all the other breech lengths, unique points of blast overpressure decays are identified along the expansion section, which is illustrated in the following section.

For L_1_, L_2_, L_3_, and L_4_, we observe the following: (i) for any membrane thicknesses, an obvious difference in blast overpressure is observed between L_1_ and other breech lengths (Figures 8A–C) (ii) beyond 3000 mm from the breech, for 5 and 10 membranes and breech length L_2_, blast overpressure starts to decay (Figures 8B,C), and (iii) beyond 5000 mm from the breech, for 10 membranes and breech lengths L_3_, the blast overpressure starts to decay (Figure 8C). Finally, for L_4_ there is no unique decay point, which implies a flat top wave occurs throughout the expansion section.

Figures 8D–F show the evolution of the PTD along the length of the shock tube driven section. For any given breech length and membrane thickness, the PTD remains reasonably constant along the length, however, decreases drastically toward the exit of the shock tube. Positive impulse is the area under the blast wave profile. Figures 8G–I show the evolution of the positive impulse along the length of the shock tube expansions section. Positive impulse is a function of both blast overpressure and PTD; hence, it increases with an increase in both membrane thickness and breech length. Owing to its relationship with the PTD, the impulse drastically reduces near the exit of the shock tube.

### Flattop or plateau wave

**Figure 9:**
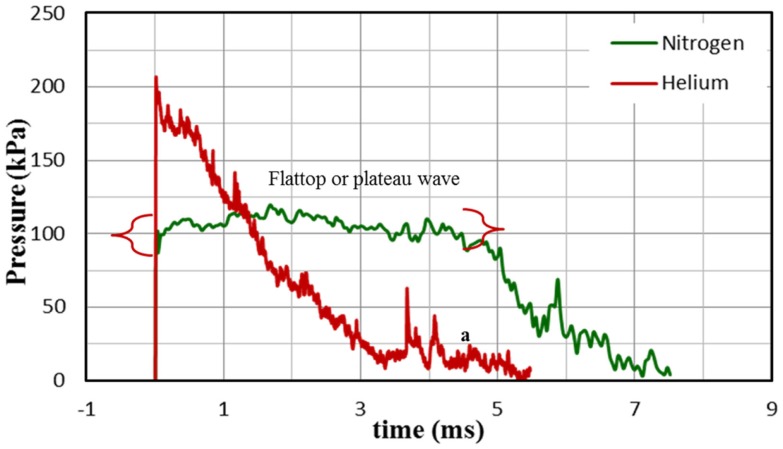
**Comparison of the shock-blast profile for helium and nitrogen with 10 membranes and breech length of 1209.68 mm; clearly the wave profile corresponding to helium gas is a Friedlander wave and wave profile corresponding to nitrogen is a flat top wave**.

A flattop or plateau wave is often observed in a gas-driven shock tube (9). In this case, the blast wave profile once reaching the peak blast overpressure maintains its peak value for a certain period of time before decay starts. Longer breech lengths in combination with the use of nitrogen as a driver gas can generate such a waveform. Figure 9 shows the comparison between the blast wave profile with nitrogen and helium as driver gas measured at test section (sensor location X). In both cases, 10 membranes with a breech length of 1209.68 mm were used. It can be seen that only in the case of nitrogen as driver gas flat top wave is observed; in the case of helium, a pure Friedlander wave is obtained.

### Comparison between field and laboratory profiles

**Figure 10:**
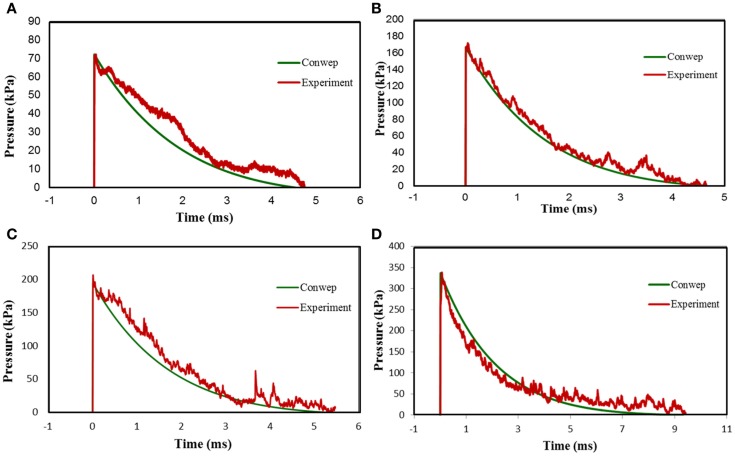
**Comparison of the shock-blast profiles from shock tube device and ConWep simulation software**. **(A)** Comparison between shock-blast profile from a 10 membrane, 66.68 mm breech length shot with nitrogen as driver gas and 2.56 kg of TNT at 5.18 m, **(B)** comparison between shock-blast profile from a 8 membrane, 752.48 mm breech length shot with helium as driver gas and 7.68 kg of TNT at 5 m, **(C)** comparison between shock-blast profile from a 10 membrane, 1209.68 mm breech length shot with helium as driver gas and 14.08 kg of TNT at 5.7 m, **(D)** comparison between shock-blast profile from 15 membrane, 1209.68 mm breech length shot with helium as driver gas and 96 kg of TNT at 8.5 m.

The primary objective of this work was to establish the shock tube parameters that can be used for generating specific blast profiles replicating field conditions. To validate this hypothesis, we compared the blast profiles of TNT explosive for different strength and range generated from ConWep with those generated from our shock tube device. Figures 10A–D show one to one comparison of the incident pressure data from shock tube test section and TNT profiles of different strength and range.

## Discussion

Experiments using animal, cadaver, or test dummies remain the foremost means to investigate injury biomechanics as well as validate numerical models, for investigative, protective, diagnostics, and therapeutic purposes. It is well recognized that input biomechanical loading experienced by the subject determines both the injury and mortality (5, 29). Hence, in the study of mild and moderate TBI, it is important to reproduce the field conditions as accurately as possible without artifacts, for offering a range of engineering solutions (such as development of better helmets) and medical strategies (such as development of better therapies and pharmaceutical regimen). In this work, we identify the essential parameters of a blast wave and describe the methodology to control them by optimizing the parameters of the laboratory gas-driven shock tube. We also identify some of the common artifacts that render the wave profile invalid in a compressed-gas-driven shock tube and discuss techniques that can be used to eliminate them.

We observed that the burst pressure does not vary significantly with respect to increase in the breech length. The rupture of the membrane is based on the contained pressure and is not affected by the volume of the gas. Thus, burst pressure that occurs at a minimum breech length L_1_ also initiates rupture at L_2_, L_3_, or L_4_. Therefore, total thickness of the membrane determines the burst pressure (Figure 4). This result corroborates with the findings from the study conducted by Payman and Shepherd, where they used copper as their membrane. They determined that for the same thickness, the burst pressure does not vary more than ±3%. Similarly, they also determined that membrane thickness has a linear relationship with burst pressure (30).

Controlling blast overpressure and PTD is essential when replicating field blasts. By manipulating breech length, burst pressure (membrane thickness), type of gas, and test section location (by varying the test section within expansion section), it was shown that it is indeed possible to obtain specified profiles. It can be seen that within test section with an increase in burst pressure, both the blast overpressure and the Mach number (strength of shock wave) increases. This implies that both these variables can be increased by increasing the membrane thickness. Similarly, PTD increases with increase in breech length for any given burst pressure. However, at lower breech lengths both blast overpressure and PTD are affected by expansion waves (also known as rarefaction waves) released from the rear end of the breech, which is explained in the next section.

**Figure 11:**
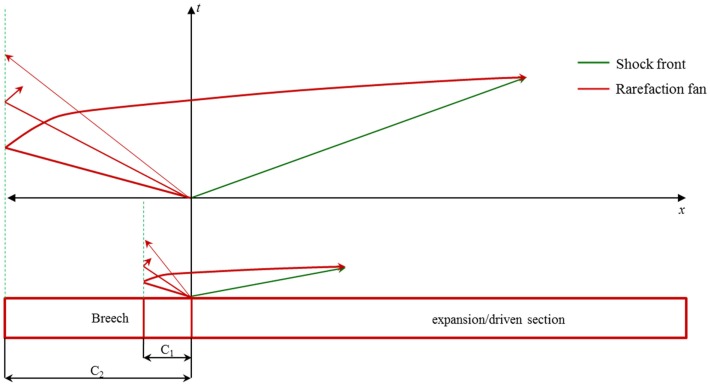
***x* − *t* Diagram for compressed-gas shock tube**. Here, a comparison was made between two driver lengths C_1_ and C_2_. Clearly, it can be seen that the expansion or rarefaction wave for C_1_ reaches earlier than C_2_.

From the *x* − *t* wave propagation diagram (Figure 11), it can be seen that the driver gas expansion initiates a family of infinite expansion waves or fan toward the closed end (rear end). Once the expansion head reaches the closed end, they are reflected and travel toward the transition. This reflected wave catches the shock front and since these waves are tensile in nature; since the shock front is compressive in nature, they start to cancel each other. With each successive expansion wave exiting the breech, the density of the gases reduces, resulting in slowing the successive expansion wave of the fan. This fan of waves arriving one after the other leads to the non-linear decay and ultimately shaping the shock-blast wave (27). Once the waveform attains the shape shown in the Figure 1B expansion waves start to erode the blast overpressure and PTD. This was observed in the behavior of waves corresponding to breech length L_1_ in the experiment, which is different from the other breech lengths. This is because for L_1_, the expansion waves almost instantaneously catch up with the shock front. For other lengths, expansion waves catches shock front further down the shock tube (downstream). Therefore, when it arrives at the test section it has already gone through some blast overpressure and PTD reduction. Once the breech length is increased, the time taken by the expansion wave to reach the shock front increases. Consequently, for breech lengths L_2_, L_3_, and L_4_ there is no change in the blast overpressure and PTD at the test section, which implies it is a flattop wave that will become a Friedlander type wave downstream (a pictorial representation is shown in Figure 11 by comparing breech lengths C_1_ and C_2_). Similar to breech length, driver gas also plays a major role in the evolution and interaction of the expansion wave. The expansion wave while traveling toward the closed end of the driver section travels with the ambient sound speed in that medium. Therefore, for a given breech length and membrane thickness, having helium (helium has a higher sound speed compared to nitrogen for any temperature) as a driver gas increases the expansion wave velocity, resulting in Friedlander type wave even at an earlier point than nitrogen gas. Consequently, by varying the length of the breech in conjunction with using the appropriate driver gas, we would be able to optimize PTD and blast overpressure.

There is an inherent relationship between SAPs; optimization of one variable might have a negative effect on the other variables, resulting in the formation of a non-optimal blast wave. This problem arises depending on (i) type of driver gas and (ii) test section location. Although nitrogen due to its low-acoustic velocity has a tendency to produce longer PTD, using a longer breech length results in a flattop wave. Conversely, helium produces a lower PTD compared to nitrogen but has a sharp decay to the atmospheric pressure (Figure 9). Similar findings are reported in literature, where they compared the wave profiles generated from air (which has acoustic velocity close to nitrogen) and nitrogen with helium. They found that using air and nitrogen as a driver gas produces a flattop wave (9, 15). One technique used for avoiding a flattop wave when using long-breech length is to place the test section downstream of the expansion section, so that the expansion waves would eventually catch up and produces a Friedlander type wave; nevertheless, this method has its own limitation, as explained in the following section.

The evolution of the blast overpressure, PTD, positive impulse at five locations along the length of the expansion section was measured. In a typical free-field blast, the blast overpressure decreases rapidly with respect to increase in distance from the blast epicenter (31). However, blast overpressure in a shock tube does not show a drastic reduction due to its constant cross section. There is a considerable difference between the blast overpressures for L_1_ and all the other breech lengths. As discussed earlier, this difference arises from the interaction of expansion waves that comes from rear end of the breech. This suggests that the expansion waves from the breech for breech length L_1_ reaches earlier than all other breech length. With increase in the breech length and burst pressure, distinct points at which the blast starts to decay are identified as shown in Figures 8B,C, which implies that downstream to this point blast wave has a Friedlander form. Consequently, for longer breech lengths that tend to produce a flattop wave upstream (e.g., test section), the wave will assume a Friedlander type wave at some point downstream. However, when moving closer to the exit the rarefaction waves from the exit starts to interact with blast wave creating artifacts, which results in inaccurate blast simulation (27). As a result, PTD reduces drastically near the exit of the shock tube due to the interaction between shock front and exit expansion waves. This has two consequences: first, the positive impulse (energy of blast wave) reduces drastically (Figures 8G–I). Second, since the total energy at the exit is conserved, all the blast energy is converted from supersonic blast wave to subsonic jet wind, which produces erroneous results (32). The effects of jet wind and specimen placement location along the expansion section for blast simulation using shock tube are illustrated in these references (2, 33).

The discussion above indicated that SAPs could be adjusted to generate a specific blast profile at a specific location (for the placement of animal model). In addition, the cross-sectional area of the specimen (animal) and specimen holder should be small compared to the entire cross-section of the tube. In all our experiments, we have found (through computer simulation and experiments) that if the specimen and holder occupy <25% of the tube area, then the reflection from the side walls do not interfere with the profile (1, 2). Further, it is always beneficial to measure the sidewall pressure just in front of the specimen and possibly one behind to get accurate loading information. A gage too far away upstream or downstream from the specimen may not yield a reliable loading data. If possible, one can actually measure the profile using surface pressure gages glued directly on the animal and this is the best choice. However, the effect of, surface orientation of the gage with respect to shock direction should also be considered.

Table 1 shows the IEDs, mine threats currently employed in the field, and their explosive capacity in TNT strength (23, 24). Using ConWep, we can determine the pressure profiles for TNT explosives within the range of strengths described in Table 1. We compared the shock tube generated wave profile with the incident wave obtained for TNT explosive in ConWep simulation software (Figure 10). Clearly, there is good match in the results (see Table 1) that indicates the wave profile generated from our shock tube can be directly related to relevant field conditions.

It may be instructive to examine how the results presented here can be applied to a variety of shock tubes currently being used in various laboratories to study blast injury animal models. What is shown in this work is to how to generate a planar shock wave that replicates mild and moderate TBI conditions when only blast (i.e., primary blast) is considered. Since blasts can be very easily produced by the sudden release of high-pressure gas, one should be careful that at the test location, the blast might not be planar. Since the planarity is achieved only after sufficient length from the membrane and away from the open end, locating specimens close to the end will result in primary plus tertiary loads (from blast wind) along with effects from the exit rarefaction wave. Thus, injury resulting from specimens kept near the open end of the tube will lead to mixed mode compared to pure primary type of loading.

## Conclusion

Compressed-gas-driven shock tubes are used by different research groups to study BINT using animal models. In order to provide field-relevant blast waves and to compare the results among different groups, it is important to know the actual shock pulse impinging on the test objects. Since the pressure-time pulse vary significantly for different explosive strength and stand-off distance it is important to tailor the blast wave parameters for a wide range in controlled and repeatable manner. This study presents how the SAPs influence SWPs. Further, the need for optimization of SAPs to avoid a flattop wave or expansion waves from the exit, are also explained. Finally, a comparison is made between wave profiles generated from a shock tube and ConWep to show that our shock tube can replicate the pressure profiles within a range of practical interest. Key findings of this work are as follows:

- Burst pressure depends only on the membrane thickness and not on the breech length (for practical ranges tested); hence, the blast overpressure increases with increase in membrane thickness. At lower breech lengths blast overpressure is affected due to the shock front interaction with expansion waves from the breech.
- Positive time duration increases with increase in the breech length; however, higher breech lengths in conjunction with use of nitrogen gas produces a plateau or a flattop wave. This problem can be solved by either using helium as driver gas or in some cases by shifting the test section downstream of expansion section (a longer shock tube may be required for avoiding exit end effects).
- When the test section is moved closer to the exit of the shock tube, the rarefaction wave from the exit creates unacceptable artifacts in the wave profile.
- From the comparison of the profiles from ConWep TNT profiles and shock tube profiles it can be concluded that compressed-gas shock tube can be used to accurately simulate primary blast injury for BINT studies.

## Conflict of Interest Statement

The authors declare that the research was conducted in the absence of any commercial or financial relationships that could be construed as a potential conflict of interest.
